# SARS-CoV-2 Infektionen und das autonome Nervensystem

**DOI:** 10.1007/s00112-021-01197-7

**Published:** 2021-04-27

**Authors:** R. Buchhorn, C. Willaschek, C. Baumann

**Affiliations:** 1 Klinik für Kinder- und Jugendmedizin, Caritas-Krankenhaus Bad Mergentheim, Bad Mergentheim, Deutschland; 2 Universität Würzburg, Würzburg, Deutschland; 3 Praxis für Kinder- und Jugendmedizin, Am Bahnhof 1, 74670 Forchtenberg, Deutschland

**Keywords:** Kinder, Multisystemisches Inflammationssyndrom, Posturales orthostatisches Tachykardiesyndrom, Herzratenvariabilität, Autoantikörper, Children, Multisystemic inflammation syndrome, Postural orthostatic tachycardia syndrome, Heart rate variability, Autoantibodies

## Abstract

Vorgestellt werden die Untersuchungen der Herzratenvariabilität (HRV) bei einem 11-jährigen Jungen mit multisystemischem Inflammationssyndrom bei Kindern (MIS-C) und einem 16-jährigen Mädchen mit einem posturalen orthostatischen Tachykardiesyndrom (POTS) jeweils nach SARS-CoV-2-Infektion. *Ergebnis: *Das MIS‑C ist durch eine maximale Suppression der HRV im EKG-Monitoring auf der Intensivstation gekennzeichnet. Nach i.v.-Immunglobulin-Gabe zeigte sich die Suppression der HRV als rasch reversibel. Das POTS ist durch einen Anstieg der Herzfrequenz um 40 Schläge/min und den Verlust der HRV im aktiven Stehtest gekennzeichnet und vermutlich eine Ursache für chronische Beschwerden nach einer SARS-CoV-2-Infektion. Bei MIS‑C konnten wir Autoantikörper gegen Rezeptoren des autonomen Nervensystems nachweisen. *Zusammenfassung: *Unsere Kasuistiken über autonome Regulationsstörungen bei Kindern mit MIS‑C und POTS nach SARS-COV-2-Infektionen sind Erstbeschreibungen, die unser Wissen zur Pathophysiologie dieser neuen Erkrankung bereichern können.

## Abstract (en)

Investigations of the heart rate variability (HRV) in an 11-year-old boy with multisystemic inflammation syndrome in children (MIS-C) and a 16-year-old girl with postural orthostatic tachycardia syndrome (POTS) after SARS-CoV‑2 infections are presented. *Results: *The MIS‑C is characterized by a maximum suppression of the HRV during ECG monitoring on the pediatric intensive care unit. After intravenous administration of immunoglobulins HRV supppression is rapidly reversible. The POTS is characterized by a heart rate increase of more than 40 bpm and the complete collapse of the HRV in the active standing test that can probably explain some chronic problems after SARS-CoV‑2 infections. In the MIS‑C patient we found autoantibodies against receptors of the autonomic nervous system. *Summary: *The examination of HRV in patients with COVID-19 are initial descriptions,which can enrich our knowledge of the pathophysiology of this new disease.

Im Mai 2020 hatten wir, basierend auf der Kasuistik einer lückenlosen Aufzeichnung der Herzratenvariabilität (HRV) während einer akuten SARS-CoV-2-Infektion (severe acute respiratory syndrome coronavirus 2) bei einem 58-jährigen Mann, auf spezifische Veränderungen der HRV hingewiesen, die auf eine Beteiligung des autonomen Nervensystems bei dieser neuen Erkrankung hinweisen [1]. Die für Infektionen typische Reduktion der HRV geht dabei bei COVID-19 (corona virus disease 2019) nicht mit einem adäquaten Herzfrequenzanstieg bei Fieber einher, der bei anderen Infektionen im Mittel 18 Schläge/min je 1 °C Temperaturanstieg beträgt [10]. Diese „relative Bradykardie“ wurde in mehreren nachfolgenden Arbeiten auf der Basis größerer Patientenzahlen bestätigt und z. T. genutzt, um mithilfe von Fitness-Trackern sowohl den Krankheitsbeginn als auch den Krankheitsverlauf [8] einer SARS-CoV-2-Infektion voraussagen zu können. So wurde in einer großen Studie gezeigt [12], dass es bei einer akuten SARS-CoV-2-Infektion nach einem frühen Anstieg der Herzfrequenz und Abfall der HRV zu einem Abfall der Herzfrequenz ab Tag 7 kommt, der seinen Höhepunkt am 15. Krankheitstag aufweist und erst ab dem 21. Tag die Ausgangswerte wieder erreicht.

Grundsätzlich kommt es im Rahmen einer Inflammation zu einem Anstieg der Herzfrequenz und einem Abfall der HRV aufgrund des sog. cholinergen antiinflammatorischen Signalweges, der 2011 in einem Review ausführlich beschrieben wurde [9]. Als pathophysiologische Erklärung für den davon abweichenden Herzfrequenzverlauf bei COVID-19 wurden eine direkte Schädigung des Sinusknoten durch die Infektion bzw. eine Infektion der regulatorischen Zentren im Bereich des Hirnstamms diskutiert [14]. Aus unserer Sicht erscheint die Hypothese der Arbeitsgruppe um Lagoumintzis et al. wahrscheinlich, dass der cholinerge antiinflammatorische Signalweg durch das SARS-CoV‑2 direkt am Acetylcholinrezeptor-7 toxisch geschädigt wird [11], was mithilfe der HRV-Analyse vermutlich frühzeitig erkannt werden kann.

Seit Dezember 2020 treten nun zunehmend gesundheitliche Probleme bei Kindern nach stattgehabter SARS-CoV-2-Infektion auf. Erneut haben wir akribisch die Veränderungen des autonomen Nervensystems bei diesen Kindern mithilfe der Analyse der HRV untersucht, um die große Bedeutung des autonomen Nervensystems hinsichtlich der COVID-19-Pandemie darstellen zu können.

## Fall 1

**Figure 1:**
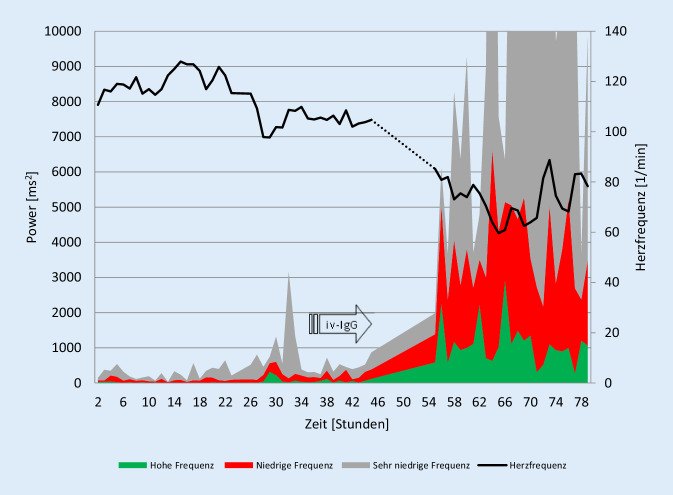

Ein 11-jähriger Junge hatte 4 Wochen vor der Vorstellung in unserer Klinik eine asymptomatische SARS-CoV-2-Infektion durchgemacht, die im Rahmen einer Untersuchung von Kontaktpersonen in der Familie mittels PCR nachgewiesen wurde. Die SARS-CoV-2-Antikörper zeigten sich entsprechend positiv. In unserer Klinik zeigte der Junge bei Aufnahme die typischen Symptome des multisystemischen Inflammationssyndroms bei Kindern (MIS-C) mit Fieber, Konjunktivitis, gastrointestinalen Beschwerden und hohen CRP- (9,4 mg/dl, Normwert < 0,5) und NT-Pro-BNP-Werten im Blut (8137 pg/ml, Normwert < 85). Die daraufhin eingeleitete Therapie mit i.v.-Immunglobulinen führte zur Entfieberung und zum Abfall des CRP (1,57 mg/dl) und NT-Pro-BNP (1108 pg/ml). Auf unserer Intensivstation nutzen wir regelmäßig die Daten des EKG-Monitorings, um über eine Netzwerkverbindung die HRV online zu analysieren. Mithilfe der Fast-Fourier-Analyse werden einzelne Frequenzbereiche der HRV dargestellt, wobei die „high frequency power“ typischerweise die Vagusaktivität repräsentiert. Das Monitoring der HRV auf der Intensivstation zeigt zu Beginn des multisystemischen Inflammationssyndroms eine maximale Reduktion der HRV (Abb. 1). Nach der eingeleiteten Behandlung mit Immunglobulinen zeigte sich rasch eine Normalisierung der HRV. Vier Tage nach der Behandlung mit Immunglobulinen konnte der Junge in gutem Allgemeinzustand entlassen werden. Die Koronararterien zeigten in mehreren Farbdopplerechokardiographien unauffällige Befunde.

Zur weiteren Klärung der schweren autonomen Regulationsstörung des MIS‑C haben wir u. a. adrenerge Autoantikörper in einem kommerziellen Labor mittels ELISA bestimmen lassen, die folgende auffällige Befunde zeigten: Anti‑α_1_-adrenerge Rezeptorantikörper 13,3 U/ml (Norm < 7 U/ml); Anti‑α_2_-adrenerge Rezeptorantikörper 18,2 U/ml (Norm < 15), antimuskarinerge cholinerge Rezeptor-3-Antikörper 14,7 U/ml (Norm < 8 U/ml), antimuskarinerge cholinerge Rezeptor-4-Antikörper 15,5 U/ml (Norm < 10,7 U/ml) und antimuskarinerge cholinerge Rezeptor-5-Antikörper 15,7 U/ml (Norm < 14,2 U/ml).

## Fall 2

Ein 16-jähriges sportliches Mädchen stellt sich mit Schwindelattacken und Präsynkopen nach dem Aufstehen vor. Sie hatte eine PCR-gesicherte SARS-CoV‑2-Infektion 14 Tage zuvor durchgemacht. Die serologischen Untersuchungen der SARS-CoV‑2-Antikörper zeigten sich auch hier entsprechend positiv. Die übliche klinische Diagnostik konnte die Symptomatik nicht klären. Im Langzeit-EKG zeigten sich keine Herzrhythmusstörung, eine normale mittlere Herzfrequenz mit 80 Schlägen/min, aber eine relevante autonome Regulationsstörung mit herabgesetzter globaler HRV (SDNN = 95 ms, Normwert > 128). Basierend auf unseren Vorarbeiten über autonome Regulationsstörungen bei Kindern [2] und ersten Fallberichten aus der internistischen Kardiologie lag es nahe [6], dass diese postinfektiösen Probleme nach der SARS-CoV-2-Infektion Ausdruck eines sog. posturalen orthostatischen Tachykardiesyndroms (POTS) sind. Dies bestätigte sich bei unserer Patientin durch einen überschießenden Herzfrequenzanstieg von mehr als 40 Schlägen im aktiven Stehtest, den sie nach nur 1 min abbrechen musste (Messung mit dem HRV-Scanner; Fa. Biosign™ GmbH, Ottenhofen, Deutschland). Das Power-Spektrum der HRV während dieser Untersuchung zeigt den fast vollständigen Verlust der HRV im Stehen als Ausdruck der schweren autonomen Regulationsstörung. Wir starteten eine niedrig dosierte β‑Blocker-Therapie mit 2‑mal 10 mg Propranolol [13], und die klinische Symptomatik war scheinbar verschwunden. Bei der Kontrolle des Stehtest kam es dann jedoch zu einer schweren vagovasalen Synkope mit einem Sinusarrest von 5 s, sodass wir die Therapie wieder beenden mussten. Die Patientin erhielt eine symptomatische Therapie mit Midodrin, unter der sie leidlich ihren Alltag bewältigt.

## Diskussion

Die Beteiligung des autonomen Nervensystems im Rahmen einer akuten SARS-CoV-2-Infektion, wie wir sie früh im Rahmen einer Kasuistik beschrieben hatten [1], kann zu Diagnostik und Verlaufskontrolle mithilfe von Fitness-Trackern genutzt werden. Die pathophysiologischen Ursachen, die methodischen Fehlermöglichkeiten und therapeutischen Konsequenzen sind komplex und können im Rahmen dieser Arbeit nicht abschließend diskutiert werden.

Offensichtlich kann es insbesondere bei Kindern zu einer schweren persistierenden autonomen Regulationsstörung nach SARS-CoV-2-Infektion im Rahmen des MIS‑C kommen, welche bei 80 % der Kinder eine intensivmedizinische Behandlung erfordert und bei 2 % tödlich verläuft [5]. Zurzeit etablieren sich, basierend auf den Erfahrungen bei der Behandlung des Kawasaki-Syndroms, Therapiestandards [4], die auch bei unseren Patienten erfolgreich waren. In unserem Fallbericht 1 konnten wir erstmals die maximale Suppression der HRV bei einem solchen Kind mit MIS‑C nachweisen, die sich unmittelbar nach der i.v.-Immunglobulin-Gabe reversibel zeigte.

Am Fallbeispiel 2 können wir zeigen, dass Kinder nach SARS-CoV-Infektion eine chronische Regulationsstörung des autonomen Nervensystems mit einem POTS entwickeln können, welches in einem einfachen aktiven Stehtest mit kontinuierlicher EKG-Registrierung nachweisbar ist. Diese Methodik des Stehtests mit HRV-Analyse hatten wir kürzlich in einer Fallsammlung mit 500 Kindern publiziert [2].

Ursächlich für derartige – oft postinfektiöse – autonome Veränderungen werden Autoantikörper gegen G‑Protein-gekoppelte Rezeptoren angenommen [7], die auch wir bei einigen der von uns untersuchten Kindern mit schweren autonomen Regulationsstörungen nachweisen konnten [2]. Im Zusammenhang mit den erhöhten adrenergen Autoantikörpern in unserem 1. Fallbeispiel erscheint es sinnvoll, die Induktion von Autoantikörpern nach SARS-COV-2-Infektion bei Kindern systematisch zu untersuchen. In einem aktuellen Review wird eindrucksvoll gezeigt, dass die SARS-CoV-2-Infektion als potenter Trigger der Bildung von Autoantikörpern angesehen wird [3].

## Fazit für die Praxis

- COVID-19, das multisystemische Inflammationssyndrom bei Kindern (MIS-C) und das posturale orthostatische Tachykardiesyndrom (POTS) nach COVID-19 sind durch Veränderungen der Herzfrequenz und Herzratenvariabilität (HRV) gekennzeichnet, die auf eine Beteiligung des autonomen Nervensystems hinweisen.
- Ursächlich werden eine Schädigung des sog. cholinergen antiinflammatorischen Signalweges und die Bildung von Autoantikörpern gegen adrenerge- und muskarinerge Rezeptoren diskutiert und in unserem Fall tatsächlich nachgewiesen.
- Wir empfehlen das Monitoring der HRV als Therapiekontrolle, wie wir es am Beispiel der i.v.-Immunglobulin-Infusion demonstrieren können.
